# Supplementary material for: SARS-CoV-2 seroprevalence and neutralizing activity in donor and patient blood
Source: Nat Commun. 2020 Sep 17;11:4698. doi: 10.1038/s41467-020-18468-8 (PMC7499171; doi:10.1038/s41467-020-18468-8)
Supplement: Supplementary file 1 — Supplementary Information [file 41467_2020_18468_MOESM1_ESM.pdf]

SUPPLEMENTARY MATERIAL

SARS-CoV-2 seroprevalence and neutralizing activity in donor and patient blood

Dianna L. Ng[1,2#], Gregory M. Goldgof[1#], Brian R. Shy[1#], Andrew G. Levine[1#], Joanna Balcerek[1#], Sagar P. Bapat[1#], John Prostko[4], Mary Rodgers[4], Kelly Coller[4], Sandra Pearce[4], Sergej Franz[3], Li Du[3], Mars Stone[1,3], Satish K. Pillai[3], Alicia Sotomayor-Gonzalez[1,5], Venice Servellita[1,5], Claudia Sanchez San Martin[1,5], Andrea Granados[1,5], Dustin R. Glasner[1,5], Lucy M. Han[1,2], Kent Truong[1,2], Naomi Akagi[1,2], David N. Nguyen[6], Neil M. Neumann[2], Daniel Qazi[2], Elaine Hsu[1], Wei Gu[1], Yale A. Santos[1,5], Brian Custer[3], Valerie Green[7], Phillip Williamson[7], Nancy K. Hills[8,9], Chuanyi M. Lu[1,10], Jeffrey D. Whitman[1], Susan L. Stramer[11], Candace Wang[1,5], Kevin Reyes[1,5], Jill M.C. Hakim[12], Kirk Sujishi[1], Fariba Alazzeh[1], Lori Pham[1], Edward Thornborrow[1], Ching-Ying Oon[1], Steve Miller[1,5], Theodore Kurtz[1], Graham Simmons[1,3&], John Hackett, Jr.[4&], Michael P. Busch[1,3&], Charles Y. Chiu[1,5,6&*]

[1]Department of Laboratory Medicine, University of California, San Francisco, San Francisco, CA, USA

[2]Department of Pathology, University of California, San Francisco, San Francisco, CA, USA

[3]Vitalant Research Institute, San Francisco, CA, USA

[4]Applied Research and Technology, Abbott Diagnostics, Abbott Park, IL, USA

[5]UCSF-Abbott Viral Diagnostics and Discovery Center, San Francisco, CA, USA

[6]Department of Medicine, Division of Infectious Diseases, University of California, San Francisco, San Francisco, CA, USA

[7]Creative Testing Solutions, Tempe, AZ, USA

[8]Department of Neurology, University of California, San Francisco, San Francisco, CA, USA

[9]Department of Epidemiology and Biostatistics, University of California, San Francisco, San Francisco, CA, USA

[10]Lab Medicine Service, San Francisco VA Healthcare System

[11]American Red Cross, Gaithersburg, MD, USA

[12]Department of Medicine at ZSFG, the Division of HIV, ID & Global Medicine

[#]These authors contributed equally to this work: Dianna L. Ng, Gregory M. Goldgof, Brian R. Shy, Andrew G. Levine, Joanna Balcerek, Sagar P. Bapat

[*]These authors jointly supervised this work: Charles Y. Chiu, Michael P. Busch, John Hackett, Jr., Graham Simmons

TABLE OF CONTENTS

Supplementary Table 1 3

Supplementary Table 1 5

Supplementary Table 1 6

Supplementary Table 1 7

Supplementary Table 1. Demographic characteristics, presenting symptoms, chronic medical conditions, medications, and radiographic findings of 43 SARS-CoV-2 PCR-positive UCSF outpatients and hospitalized patients.

**Clinical characteristic**	**Positive** (n=43)
Age (years)	59±18
Male	28 (65.1%)
Female	12 (27.9%)
Other/Unknown	3 (6.98%)
**Racial or ethnic group**	
Hispanic/LatinX	8 (18.6%)
Asian	7 (16.3%)
White	12 (27.9%)
Black	2 (4.65%)
Other/not reported	14 (32.6%)
**Smoking status**	
Never smoker	19 (44.2%)
Former smoker	11 (25.6%)
Current smoker	1 (2.33%)
Unknown/not reported	11 (25.6%)
**Presenting symptoms**	
Cough	32 (74.4%)
Fever	27 (62.8%)
Myalgia	15 (34.9%)
Shortness of breath/Dyspnea	19 (44.2%)
Chest pain	7 (16.3%)
Headache	8 (18.6%)
Chills	9 (20.9%)
Sore throat	7 (16.3%)
Malaise/fatigue	21 (48.8%)
Diarrhea	10 (23.3%)
Anorexia	5 (11.6%)
Nausea and/or vomiting	5 (11.6%)
Anosmia and/or dysgeusia	2 (4.65%)
Asymptomatic	2 (4.65%)
Not documented	3 (6.98%)

Chronic medical conditions	
Hypertension	16 (37.2%)
Type 2 Diabetes Mellitus	14 (32.6%)
Hyperlipidemia	10 (23.3%)
Obesity	9 (20.9%)
Chronic Kidney Disease	8 (18.6%)
Hypothyroid	7 (16.3%)
Solid organ transplant	6 (14.0%)
Coronary Artery Disease	7 (16.3%)
Asthma	3 (6.98%)
Autoimmune Disease	2 (4.65%)
Congestive Heart Failure	4 (9.30%)
Liver disease	1 (2.33%)
Malignancy	2 (4.65%)
Emphysema/COPD	0 (0%)
Prior stroke	4 (9.30%)
HIV	0 (0%)
No co-morbidities	2 (4.65%)
Medical history not documented	6 (14.0%)
**Medications**	
ACE inhibitor use	5 (11.6%)
Chemotherapy	0 (0%)
Immunosuppressive agents	7 (16.3%)
Remdesivir (or placebo)	12 (27.9%)
Hydroxychloroquine	6 (14.0%)
Corticosteroids	2 (4.65%)
Not documented	9 (20.9%)
**Severity**	
Ambulatory*	8 (18.6%)
Admitted	29 (67.4%)
ICU	15 (34.9%)
Not documented	1 (2.33%)
**Radiographic findings, chest X-ray or computed tomography**	
Unilateral infiltrates	2 (4.65%)
Bilateral infiltrates	22 (51.2%)
Ground glass opacities	11 (25.6%)
Consolidation	11 (25.6%)
Normal imaging	5 (11.6%)
Imaging not performed	5 (11.6%)
Not documented	6 (14.0%)

*Ambulatory care includes outpatient and patients seen in ED and not admitted.

Supplementary Table 2. Demographic characteristics of individuals who donated blood at San Francisco Bay Area community blood centers (Vitalant Research Institute).

**Reported sex**	Total (n=1000)
Male	508
Female	491
Other/Unknown	
**Age (years)**	
≤19	18
20-29	152
30-39	205
40-49	172
50-59	185
60-69	197
70-79	63
80-89	7
**Race or ethnic group**	
White	686
Asian	99
Latino	90
Black	15
Other/Unknown	110

Supplementary Table 3. Demographic characteristics of UCSF patients with positive SARS-CoV-2 RT-PCR, negative SARS-CoV-2 RT-PCR, and patients who were not clinically suspected to have SARS-CoV-2 infection.

	**SARS-CoV-2 RT-PCR Positive (n=43)**	**SARS-CoV-2 RT-PCR Negative (n=163)**	**SARS-CoV-2 RT-PCR Not Performed (n=387)**
**Reported sex**			
Male	28 (65.1%)	78 (47.9%)	172 (44.4%)
Female	12 (27.9%)	84 (51.5%)	186 (48.1%)
Other/Unknown	3 (6.98%)	1 (0.6%)	29 (7.49%)
**Age (years)**			
≤19	0 (0%)	5 (3.1%)	15 (3.88%)
20-29	1 (2.3%)	11 (6.7%)	28 (7.23%)
30-39	7 (16.3%)	16 (9.8%)	41 (10.6%)
40-49	7 (16.3%)	23 (14.1%)	54 (14.0%)
50-59	9 (20.9%)	31 (19.0%)	68 (17.6%)
60-69	3 (7.0%)	40 (24.5%)	71 (18.3%)
70-79	10 (23.3%)	21 (12.9%)	56 (14.5%)
80-89	4 (9.3%)	8 (4.9%)	20 (5.17%)
90-99	2 (4.7%)	7 (4.3%)	5 (1.29%)
Unknown	0 (0%)	1 (0.6%)	29 (7.5%)
**Race or ethnic group**			
White	8 (18.6%)	74 (45.4%)	165 (42.6%)
Asian	7 (16.3%)	31 (19.0%)	85 (22.0%)
Latinx	12 (27.9%)	15 (9.2%)	37 (9.56%)
Black	2 (4.65%)	25 (15.3%)	36 (9.30%)
Other/Unknown	14 (32.6%)	18 (11.0%)	64 (16.5%)

Supplementary Table 4. Association of IgG, IgM, and neutralizing antibody (NT80) titers with disease severity in COVID-19 patients.

	Not severe		Severe (ICU/death)		p-value*
	median	(IQR)	median	(IQR)	
IgG					
Week 1	0.05	(0.02, 1.75)	1.165	(0.025, 1.35)	0.80
Week 2	2.44	(0.1875, 3.795)	2.11	(1.50, 4.72)	0.38
Week 3	2.475	(0.085, 5.315)	5.04	(4.76, 5.54)	0.07
Week4	3.965	(1.42, 5.23)	5.33	(4.90, 5.81)	0.05
IgM					
Week 1	0.155	(0.03, 1.065)	0.055	(0.02, 9.705)	0.75
Week 2	2.17	(0.11, 4.975)	6.31	(1.12, 10.4)	0.34
Week 3	1.36	(0.12, 12.4)	13.0	(6.84, 15.0)	0.067
Week4	4.88	(1.39, 8.53)	10.9	(6.42, 17.8)	0.067
NT80					
Week 1	41.8 (n=3)	(40, 73.0)	40 (n=3)	(40.0, 135)	1.00
Week 2	91.8 (n=5)	(67.3, 328)	143 (n=8)	(40.0, 216)	0.86
Week 3	94.9 (n=5)	(40, 2041)	1330 (n=3)	(57.7, 3557)	0.53
Week4	184 (n=6)	(90.5, 471)	1895 (n=6)	(669, 4825)	0.18

*p-values were calculated using the Wilcoxon Rank Sum Test
